# Supplementary figures and images for: Long-term molecular turnover of actin stress fibers revealed by advection-reaction analysis in fluorescence recovery after photobleaching
Source: PLoS One. 2022 Nov 7;17(11):e0276909. doi: 10.1371/journal.pone.0276909 (PMC9639824; doi:10.1371/journal.pone.0276909)

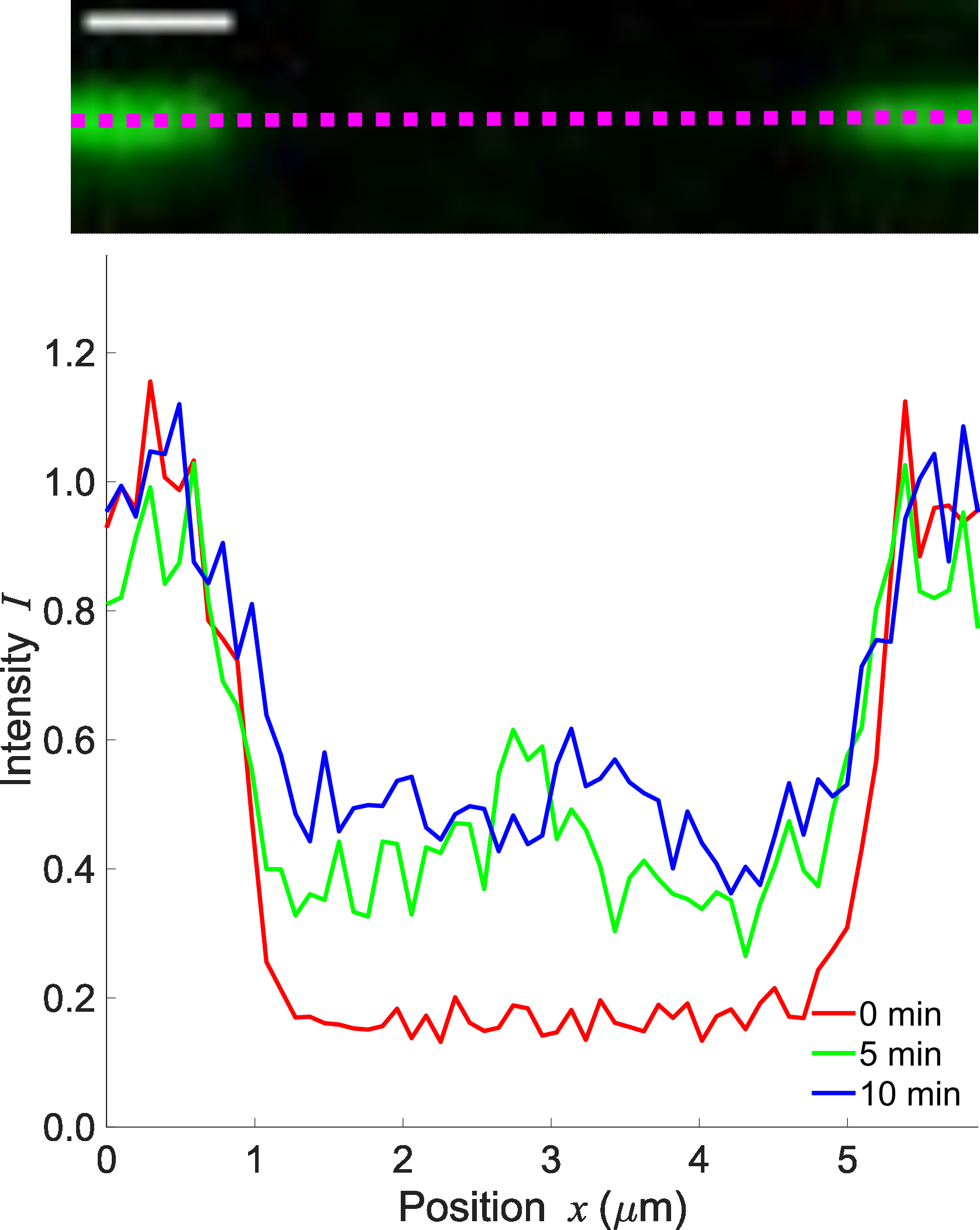

Supplement: S1 Fig — Scale, 1 μm. (TIFF) [file pone.0276909.s001.tiff]

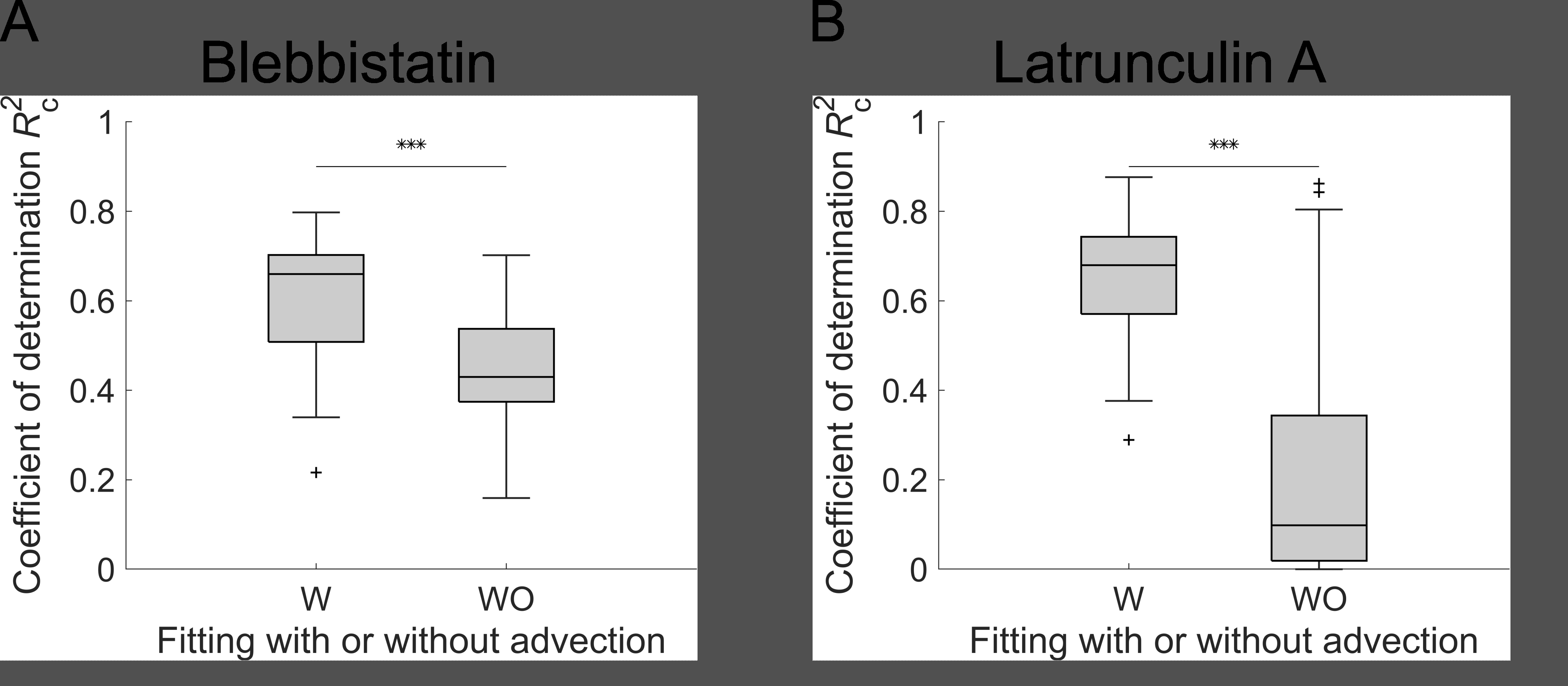

Supplement: S2 Fig — (TIFF) [file pone.0276909.s002.tiff]
