# Supplementary material for: Long-term molecular turnover of actin stress fibers revealed by advection-reaction analysis in fluorescence recovery after photobleaching
Source: PLoS One. 2022 Nov 7;17(11):e0276909. doi: 10.1371/journal.pone.0276909 (PMC9639824; doi:10.1371/journal.pone.0276909)
Supplement: S1 File — (DOCX) [file pone.0276909.s008.docx]

$$\begin{aligned} \frac{\partial C}{\partial t}=-k_{off}C+{k'}_{on}F_{eq}-U\nabla C\#\left( 1 \right) \end{aligned}$$

$$\begin{aligned} \frac{\partial C}{\partial t}=-k_{off}C+{k^{'}}_{on}F_{eq}\#\left( 2 \right) \end{aligned}$$

$$\begin{aligned} C(x,y,t)=C_{eq}(x,y)+Ae^{-k_{off}t}\#\left( 3 \right) \end{aligned}$$

$$\begin{aligned} C(x,y,t)=C_{eq}(x,y)\left( 1-e^{-k_{off}t} \right)+\phi\left( x,y \right)e^{-k_{off}t}\#\left( 4 \right) \end{aligned}.$$

$$\begin{aligned} C(x,y,t)=C_{eq}\left( x-U_{x}t,y-U_{y}t \right)\left( 1-e^{-k_{off}t} \right)+\phi\left( x-U_{x}t,y-U_{y}t \right)e^{-k_{off}t}\#\left( 5 \right) \end{aligned}$$

$$\psi\left( x,y,t \right)=1-\frac{K_{0}}{4}\left[ erf \left( \frac{x+\frac{L_{x}}{2}}{\sqrt{R^{2}+4D_{eff}t}} \right)-erf \left( \frac{x-\frac{L_{x}}{2}}{\sqrt{R^{2}+4D_{eff}t}} \right) \right]$$

$$\begin{aligned} \left[ erf \left( \frac{y+\frac{L_{y}}{2}}{\sqrt{R^{2}+4D_{eff}t}} \right)-erf \left( \frac{y-\frac{L_{y}}{2}}{\sqrt{R^{2}+4D_{eff}t}} \right) \right]\#(6) \end{aligned}$$

$$\psi\left( x,y \right)=1-\frac{K_{0}}{4}\left[ erf \left( \frac{x+\frac{L_{x}}{2}}{R} \right)-erf \left( \frac{x-\frac{L_{x}}{2}}{R} \right) \right]$$

$$\begin{aligned} \left[ erf \left( \frac{y+\frac{L_{y}}{2}}{R} \right)-erf \left( \frac{y-\frac{L_{y}}{2}}{R} \right) \right]\#(7) \end{aligned},$$

$$\begin{aligned} C_{eq}\left( x,y \right)=\frac{1}{2}\left[ erf \left( \frac{y+\frac{W}{2}}{R} \right)-erf \left( \frac{y-\frac{W}{2}}{R} \right) \right]\#\left( 8 \right) \end{aligned}$$

$$\begin{aligned} \phi\left( x,y \right)=C_{eq}\left( x,y \right)\psi\left( x,y \right)\#\left( 9 \right) \end{aligned}.$$

$$\begin{aligned} \frac{\partial C}{\partial t^{*}}=-C+\frac{{k'}_{on}}{k_{off}}F_{eq}-\frac{U}{k_{off}L_{sf}}\nabla^{*}C=-C+C_{eq}-\alpha\nabla^{*}C\#\#(10) \end{aligned}$$

$$\begin{aligned} \alpha=\frac{U}{L_{sf}k_{off}}\#\left( 11 \right) \end{aligned}.$$

$$\begin{aligned} r=\frac{\left| N_{sf}\cdot U \right|}{\left| N_{sf} \right|\left| U \right|}\#\left( 12 \right) \end{aligned}$$

$$\frac{\partial F}{\partial t}=k_{off}C-k_{on}FS$$

$$\frac{\partial S}{\partial t}=k_{off}C-k_{on}FS$$

$$\frac{\partial C}{\partial t}=-k_{off}C+k_{on}FS-U\nabla C$$

$$C_{i,j}^{n+1}=C_{i,j}^{n}+\Delta t^{*}\left( C_{eq}^{n}-C_{i,j}^{n} \right)$$

$$-\frac{\nu+\left| \nu\right|}{2}\left( C_{i,j}^{n}-C_{i-1,j}^{n} \right)-\frac{\nu-\left| \nu\right|}{2}\left( C_{i+1,j}^{n}-C_{i,j}^{n} \right)$$

$$-\frac{\nu+\left| \nu\right|}{2}\left( C_{i,j}^{n}-C_{i,j-1}^{n} \right)-\frac{\nu-\left| \nu\right|}{2}\left( C_{i,j+1}^{n}-C_{i,j}^{n} \right)$$

**Supplementary materials**

**Detailed derivation of the advection-reaction model**

To obtain eq. (1), we describe a set of original reaction-diffusion equations

$$\frac{\partial F}{\partial t}=k_{off}C-k_{on}FS$$

$$\frac{\partial S}{\partial t}=k_{off}C-k_{on}FS$$

$$\frac{\partial C}{\partial t}=-k_{off}C+k_{on}FS-U\nabla C$$

where $F, S$, and $C$ represent the concentration of free actin molecules, binding sites, and complexes, respectively, and $k_{\mathrm{on}}$ and $k_{\mathrm{off}}$ represent the association and dissociation rates, respectively. Now, we consider the following two assumptions. Since photobleaching does not change the number of binding sites, its concentration is considered to be at equilibrium, i.e., $S=S_{\mathrm{eq}}$. It turns out that pseudo-first-order rate is described by ${k'}_{\mathrm{on}}=k_{\mathrm{on}}S_{\mathrm{eq}}$. Second, the free actin molecules are provided from the free actin molecular pool, which is known to be a “house-keeping” gene. Therefore, the concentration of free actin molecules is also at equilibrium, reducing to $C_{\mathrm{eq}}={F_{\mathrm{eq}}{k'}_{\mathrm{on}}}/{k_{\mathrm{off}}}$. The normalized intensity yields $1 (=F_{\mathrm{eq}}+C_{\mathrm{eq}})$, and hence $C_{\mathrm{eq}}={{k'}_{\mathrm{on}}}/\left( {k'}_{\mathrm{on}}+k_{\mathrm{off}} \right)$. Consequently, the advection-reaction equation is reduced to a partial differential equation of the complexes as rewritten in eq. (1).

**The first-order upwind scheme for the numerical calculation**

To numerically calculate the advection-reaction equation, the first-order upwind scheme was used as follow:

$$C_{i,j}^{n+1}=C_{i,j}^{n}+\Delta t^{*}\left( C_{eq}^{n}-C_{i,j}^{n} \right)$$

$$-\frac{\nu+\left| \nu\right|}{2}\left( C_{i,j}^{n}-C_{i-1,j}^{n} \right)-\frac{\nu-\left| \nu\right|}{2}\left( C_{i+1,j}^{n}-C_{i,j}^{n} \right)$$

$$-\frac{\nu+\left| \nu\right|}{2}\left( C_{i,j}^{n}-C_{i,j-1}^{n} \right)-\frac{\nu-\left| \nu\right|}{2}\left( C_{i,j+1}^{n}-C_{i,j}^{n} \right)$$

where $\nu=\alpha/{\Delta{x^{*}}^{2}}$, and $n, i$, and $j$ represent the iteration of calculation, position along x, and that of y, respectively.
